# Full-Thickness Skin Graft according to Surrounding Relaxed Skin Tension Line Improves Scar Quality in Facial Defect Coverage: A Retrospective Comparative Study

**DOI:** 10.1155/2021/7398090

**Published:** 2021-09-15

**Authors:** Jeonghwan Shin, Uk Jang, Sang Oon Baek, Jun Yong Lee

**Affiliations:** ^1^Daesung Medical Center, Republic of Korea; ^2^Department of Plastic and Reconstructive Surgery, College of Medicine, The Catholic University of Korea, Seoul, Republic of Korea

## Abstract

A full-thickness skin graft (FTSG) is useful for covering small skin and soft tissue defects. In this paper, we suggest FTSG in consideration of the relaxed skin tension line (RSTL) concept for scar quality improvement since FTSG has disadvantages, including contour irregularities and mismatches of color and texture. We conducted a retrospective chart review of twenty-one patients with skin cancer on the face who underwent wide excision and FTSG by a single surgeon from October 2013 to July 2019. Twenty-one patients with skin cancer on the face were divided into RSTL-matched and RSTL-unmatched groups, and FTSG was performed. Each group was subjected to scar assessment three months after surgery. Observer assessment was performed by five independent observers using the observer component of the patient and observer scar assessment scale (POSAS) and Vancouver scar scale (VSS). Our results indicate that there were significant differences between the RSTL-matched and RSTL-unmatched groups in the VSS and POSAS components. In addition, the RSTL-matched group showed a natural appearance with surrounding tissue in the dynamic animation phase compared to the unmatched group. RSTL-matched FTSG can be an attractive option for face skin and soft tissue defect coverage. (An earlier version of this paper has been presented at the International Conference on PRS Korea 2020.)

## 1. Introduction

Skin grafting is the gold standard for covering large-area skin defects when there are partial-thickness defects with intact underlying musculatures [1]. A skin graft can be performed faster than a local flap, and it has the advantage that it has less donor site morbidity and can be repeatedly implemented. However, there are functional and esthetic disadvantages such as wound contracture and color mismatch in skin grafts [2]. Full-thickness skin grafts (FTSG) have been developed to overcome these limitations. When the subdermal fat layer was removed and skin harvesting performed with a thickness of 0.6 mm or more, contracture was significantly reduced compared to the thinly harvested skin graft, and a significant esthetic outcome was guaranteed by providing thickness and skin appendage to the recipient site [3–5]. Moreover, to overcome the mismatch of texture and color, full-thickness skin was harvested through the donor site adjacent to the defect area [6]. Nevertheless, the mismatch of the skin texture around the grafts and defects made scarring of the graft margins stand out and unnatural results when the graft was taken.

When making an incision in the skin, the operator applies the incision considering the relaxed skin tension line (RSTL). This action ensures cosmetic and functional results by securing minimal tension when closing defects [7]. With this in mind, we thought there would be significant esthetic results when the RSTL of the FTSG was placed parallel to the RSTL of the recipient site.

The main purposes of the present retrospective comparative study are to demonstrate the esthetic outcome and satisfaction of skin graft through RSTL matching from the perspective of patients and observers.

## 2. Patients and Methods

The Catholic Medical Center institutional review board approved our study (IRB OC20RASI0108). A retrospective chart review of twenty-one patients who underwent wide excision of facial skin cancer and immediately underwent a FTSG on the facial skin defect was conducted at the Catholic Medical Center in Korea over a 7-year period (2013-2019).

**Figure 1:**
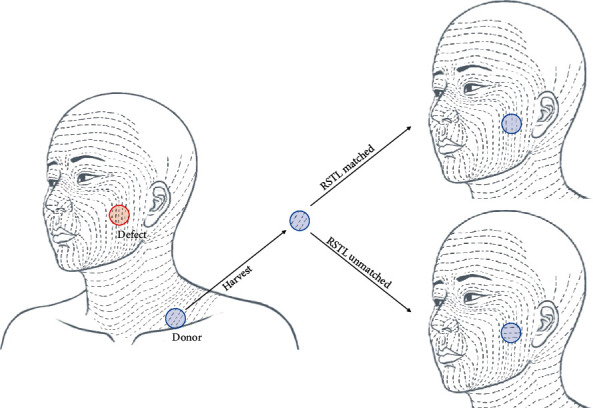
The schematic illustration of study design. We performed a wide excision of facial skin cancer and immediately performed a FTSG on the facial skin defect. Patients were divided into groups that underwent FTSG matching RSTL between donor and recipient and those who did not follow RSTL. In the RSTL-matched group, the graft was harvested at the donor site, trimmed according to the defect, and applied to facial defects in accordance with RSTL. In the RSTL-unmatched group, the harvested graft was applied to the facial defect regardless of the RSTL of the donor graft.

We performed a wide excision of facial skin cancer and immediately performed a FTSG on the facial skin defect. Patients were divided into groups that underwent FTSG matching RSTL between donor and recipient and those who did not follow RSTL (Figure 1). In the RSTL-matched group, the graft was harvested at the donor site, trimmed according to the defect, and applied to facial defects in accordance with RSTL. In the RSTL-unmatched group, the harvested graft was applied to the facial defect regardless of the RSTL of the donor graft. Finally, the graft was fixed in a tie-over manner using #5-0 black silk. The donor site was closed primarily. A scar assessment of the patient and the observer was performed three months after the operation. The scar was evaluated using two types of assessment tools. Results were analyzed according to the assessment tool, observer, and patient. Thereafter, we tried to determine the difference in scar assessment between patients and observers by comparing the RSTL-matched and RSTL-unmatched groups.

The decision to match RSTL was made intraoperatively and by the attending plastic surgeon. Since the surgical outcome of FTSG may vary by surgical technique, infection, complications, and so on, all surgeries were performed by a single senior surgeon (JY Lee) to prevent these problems, and there was no difference in the procedure in the two groups except for the grafted skin's RSTL orientation. In addition, as a problem occurred in grafts taken, cases requiring secondary procedures such as debridement were not included in this study.

Data on patient age, sex, defect area, defect site, donor site, and type of skin cancer were obtained. Each patient was surveyed for patient components of the patient and observer scar assessment scale (POSAS) three months after surgery. At the same time, scar assessment was conducted by five observers (plastic surgeons) using the POSAS and Vancouver scar scale (VSS).

### 2.1. Patient and Observer Scar Assessment Scale

The POSAS is a scar assessment tool developed in 2004 and has both an observer and a patient scar rating system [8]. The observer scar assessment scale (OSAS) was rated through five components (vascularity, pigmentation, thickness, relief, and pliability) in the original version, and in the modified version, two components (surface area, overall opinion) were added and used [9]. The patient scar assessment scale (PSAS) was rated through 6 components (pain, itching, color, stiffness, thickness, and irregularity). Each component is rated with a 10-point scoring system, with a score of 1 for normal skin and 10 for the worst imaginable scar or sensation (Supplementary 1).

### 2.2. Vancouver Scar Scale

Introduced in 1990, the VSS was the first validated scar scale to be adopted in clinical practice for the assessment of burn scars and remains one of the most frequently used scales to date [10]. Scars are assessed based on four variables: pigmentation, vascularity, pliability, and height. Scores are then assigned across these four variables based on the degree of variance from normal skin (Supplementary 2).

### 2.3. Surgical Technique

First, the RSTL of the skin cancer site was marked. Wide excision for skin cancer was performed considering RSTL. The margin was determined between 3 and 6 mm considering the skin cancer type and patient risk. All defects were dissected into the subcutaneous layer. The donor site was selected as one of the posterior auricular area and the supraclavicular area considering the skin texture and color of the defect site. The RSTL of the donor site was then marked. In the RSTL-matched group, the graft was harvested at the donor site, trimmed according to the defect, and applied to facial defects in accordance with RSTL. In the RSTL-unmatched group, the harvested graft was fixed to the facial defect regardless of the RSTL of the donor graft. Finally, the graft was fixed in a tie-over manner using #5-0 black silk. The donor site was closed primarily.

### 2.4. Statistical Analysis

First, the intraclass correlation coefficient (ICC) of the component of the VSS and observer component of the OSAS was measured to check the reliability between the five observers. In other literature, an ICC within the range of 0 to 0.20 was considered as “slight,” 0.21 to 0.40 as “fair,” 0.41 to 0.60 as “moderate,” 0.61 to 0.80 as “substantial,” and 0.81 to 1.0 as “almost perfect” [11]. The correlation between the patient component of the POSAS and the observer component of the VSS and the POSAS was evaluated using Pearson's correlation statistics.

The scar assessment scale difference between the groups that underwent FTSG according to RSTL and the group that underwent FTSG regardless of RSTL was analyzed using the Mann–Whitney *U* test. Statistical significance was set at *p* < 0.05. Statistical analyses were performed using PASW statistics 26 (IBM, Armonk, NY, USA; formerly SPSS statistics).

## 3. Result

From October 2013 to July 2019, 21 patients (5 males and 16 females) were included. The average age was 75.9 years. There were nine patients (42.9%) in the RSTL-matched group and 12 (57.1%) in the RSTL-unmatched group. The skin cancer types were basal cell carcinoma (*n* = 9), squamous cell carcinoma (*n* = 6), and Bowen's disease (*n* = 6). The defect sites were the cheek (*n* = 6), nose (*n* = 10), philtrum (*n* = 1), and temple (*n* = 4), while donor sites were postauricular (*n* = 7) and supraclavicular (*n* = 14) areas (Table 1).

### 3.1. Interobserver Reliability

Interobserver reliability was almost perfect in total (0.854) of the VSS and vascularity (0.822), overall (0.837), and total (0.823) of the observer component of the POSAS. In the VSS, pigmentation (0.568) was fair, and pliability (0.678) and height (0.637) were substantial. In the OSAS, pigmentation (0.787), thickness (0.686), relief (0.658), pliability (0.764), and surface area (0.781) were substantial (Table 2).

### 3.2. Correlation between the Patient Component of the POSAS (PSAS) and the Observer Component of the POSAS (OSAS) and VSS

Among the patient components of the POSAS (PSAS), color was matched with the VSS component of vascularity and pigmentation and with the POSAS observer component (OSAS) of vascularity and pigmentation. PSAS stiffness was matched with the VSS component of pliability and the OSAS component of pliability. PSAS thickness was matched with the VSS component of height and OSAS component of thickness; PSAS irregularity and overall were matched with OSAS component of relief and overall, respectively [12]. Among these, PSAS stiffness vs. VSS pliability, PSAS thickness vs. VSS height, and PSAS total vs. VSS total had significant results (Table 3).

### 3.3. VSS Comparison according to the RSTL

The RSTL-matched and RSTL-unmatched groups showed significant differences in all components of the VSS. The average VSS component of “total” in the RSTL-matched group was 1.78 and that in the RSTL-unmatched group was 3.98, with a *p* value of ≤0.001. Similarly, the *p* value of each component was significantly different from “pigmentation” (0.023), “vascularity” (0.007), “pliability” (≤0.001), and “height” (0.049) (Table 4).

### 3.4. Observer Scar Assessment Scale Comparison according to RSTL

There were significant differences between the RSTL-matched and RSTL-unmatched groups in the OSAS component. The average OSAS component of “total” in the RSTL-matched group was 10.98 and that in the RSTL-unmatched group was 14.49, and there was a significant difference with a *p* value of 0.002. In addition, significant differences were found in “pliability” (0.002), “surface area” (0.002), and “overall” (0.003). There were no significant differences in “vascularity” (0.464), “pigmentation” (0.082), “thickness” (0.148), and “relief” (0.095) (Table 5).

### 3.5. Patient Scar Assessment Scale (PSAS) Comparison according to RSTL

There were significant differences between the RSTL-matched and RSTL-unmatched groups in the PSAS component. The average of the PSAS component of “total” in the RSTL-matched group was 8.89 and that in the RSTL-unmatched group was 13.92, with a significant difference (≤0.001). In addition, significant differences were found in “stiffness” (*p* = 0.009), “thickness” (*p* ≤ 0.001), “irregularity” (*p* = 0.007), and “overall” (*p* = 0.012). There were no significant differences in “painful” (*p* = 0.111), “itching” (*p* = 0.702), and “color” (*p* = 0.917) (Table 6).

## 4. Discussion

FTSG can be a simple and esthetic reconstruction option when used correctly. FTSG has a short learning course and can be performed without any burden. In addition, because it does not distort the surrounding structures, the surrounding tissue can be preserved for later [2]. However, full-thickness skin may look outstanding due to a mismatch of skin color and texture of donor and recipient sites. To reduce this mismatch of the grafted skin, surgeons use donor sites with similar skin color and texture, which is essential in static conditions. Since the skin repeatedly stretches and contracts according to the movement, we suspect that matching the dynamic component by matching the RSTL would make the result better. It was also suggested that if the dynamic components were matched, there would be a significant difference between wound healing and marginal scar formation.

According to previous literature, RSTL shows a parallel arrangement of collagen bundles of the dermal layer when intrinsic tension is made [13, 14]. In order to avoid a possibly disordered arrangement of collagen fibers in the scarring wound [15], these collagen bundles must be aligned in the same direction to reduce scarring.

In the VSS comparison according to RSTL, the RSTL-matched and unmatched groups showed significant differences in all components, whereas in the OSAS comparison according to RSTL, there were only significant differences in four components: “pliability,” “surface area,” “overall,” and “total.” Unlike all components in OSAS that were graded on a ten-point scale, the VSS is represented by 0 to 3 points for “vascularity,” 0 to 2 points for “pigmentation,” 0 to 5 points for “pliability,” and 0 to 3 points for “height.” In addition, the VSS has 4 components, and the OSAS has 7 components. In fact, “vascularity,” “pigmentation,” and “thickness,” which had no significant difference in OSAS, were expressed as 0 to 2 points and 0 to 3 points in the VSS, so the interval between scores can be said to be relatively large compared to those in the OSAS.

**Figure 2:**
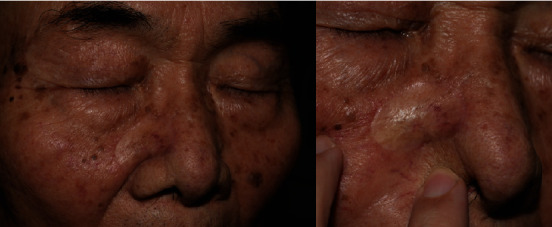
Photograph taken three months after the RSTL-matched FTSG. The patient underwent a FTSG according to RSTL after wide excision for BCC on the right cheek. The patient gave 1 point for the “thickness,” and the score was lower than the overall average (2.0). However, the observer gave 2 points for the “thickness,” and the score was higher than the average (1.84), and the observer gave 1 point for the “surface area,” and the score was lower than the average (2.37). The observer component of “thickness” was not significant, and “surface area” was significant. These two observer components are expressed as a single component called “thickness” to the patient. Therefore, this resulted in the patient's “thickness” being accepted as a meaningful result.

In the PSAS comparison according to RSTL, there were significant differences in four components: “stiffness,” “thickness,” “irregularity,” and “overall.” This should be considered in comparison to the results of the OSAS comparison according to RSTL. PSAS stiffness can be matched to OSAS pliability. PSAS irregularity can be matched to OSAS relief, and overall PSAS can be matched to overall OSAS [12]. However, the thickness, which had a significant difference in the patient (Table 6), was not significant in the observer (Table 5). The reason may be the thickness of the patient reflects a mixture of thickness and evaluation of the surface area when touched. Indeed, the evaluation of the surface area of the observer was significant. For example, Figure 2 shows a picture of a patient who underwent a FTSG according to RSTL after wide excision for BCC on the right cheek. The patient gave 1 point for the “thickness,” and the score was lower than the overall average (2.0). However, the observer gave 2 points for the “thickness” and the score was higher than the average (1.84), and the observer gave 1 point for the “surface area” and the score was lower than the average (2.37). When the patient's “thickness” included the observer's “thickness” and the “surface area,” the “thickness” was not significant in the observer, but the “surface area” was significant. This resulted in the patient's “thickness” being accepted as a meaningful result.

It is very difficult to obtain an esthetic outcome due to dimpling scar formation when performing graft in deep wounds, and in this case, the local flap could often be more meaningful [16]. Also, local flaps may be considered the reconstructive option for small- to moderate-sized defects of the face [17–19]. Although a FTSG is difficult to secure sufficient thickness and requires a well-vascularized wound bed with intact underlying structures, if the graft that was performed on the wound bed met these conditions, FTSG could be a good reconstruction option of the facial defect. We limited FTSG to facial skin cancer in this study, since skin cancer generally does not injure the underlying musculature when performing wide excision. From the patient's point of view, some patients felt a lot of pressure about the increase in the surgical range and the length of the scar when the local flap was applied. In the case of using FTSG, even if skin color discrepancy occurred immediately after surgery, the difference was reduced over time, so the satisfaction was high.

**Figure 3:**
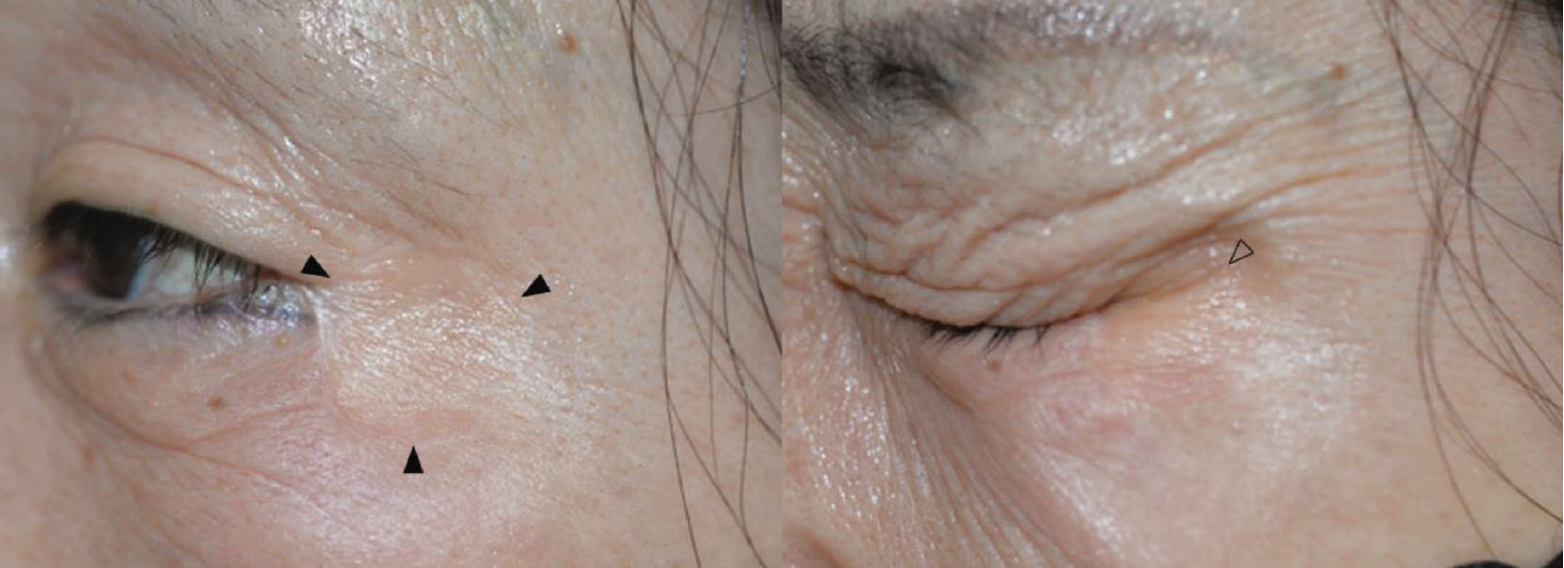
Photograph taken two years after the RSTL-matched FTSG. Black arrow indicates graft site. The empty arrow shows natural wrinkles during facial animation.

**Figure 4:**
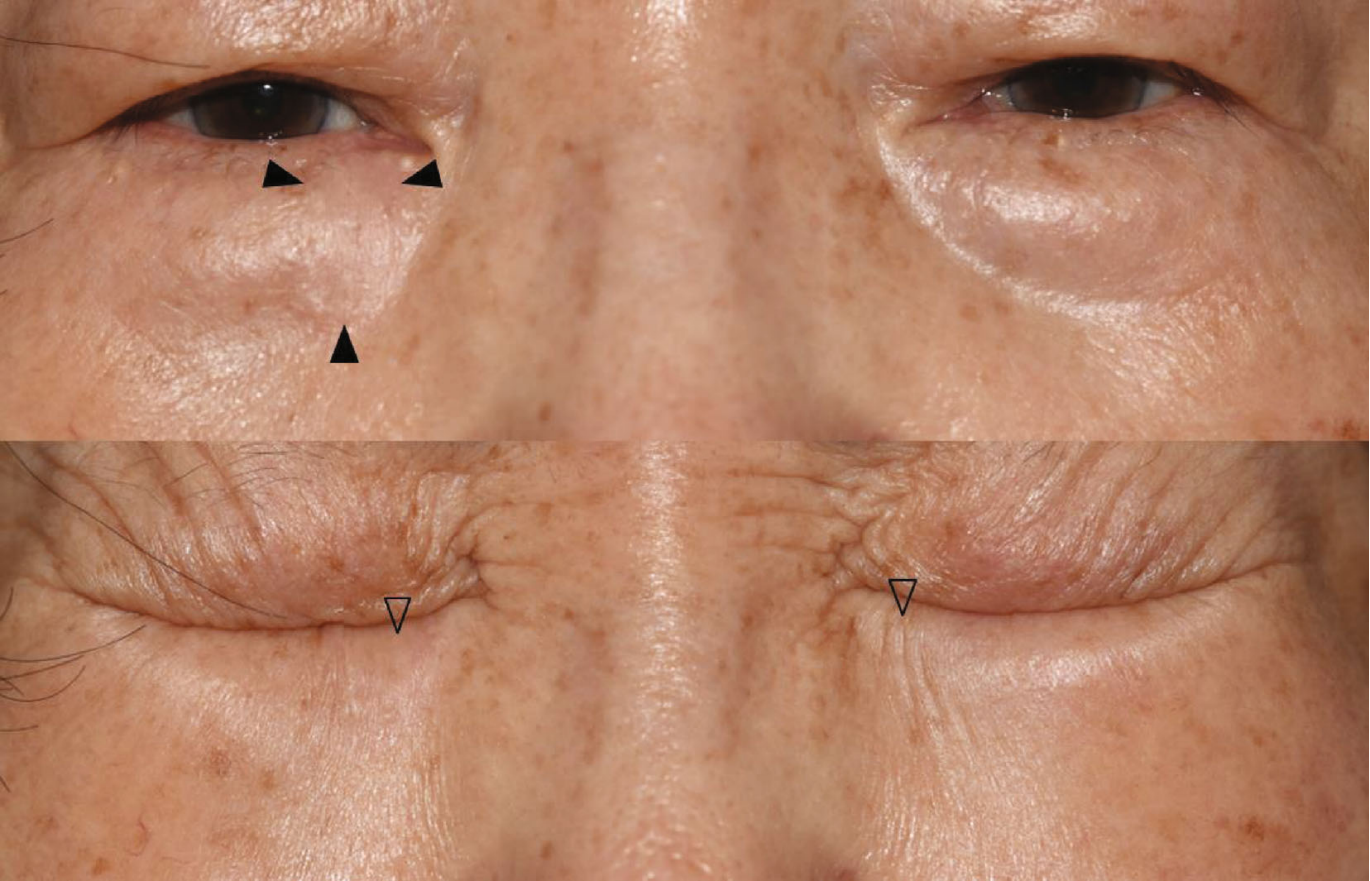
Photograph taken two years after the RSTL-unmatched FTSG. Black arrow indicates graft site. When comparing the empty arrows on both sides, the graft site does not clearly show wrinkle formation.

The RSTL-matched group showed a natural appearance with surrounding tissue in the dynamic animation phase compared to the unmatched group.  Figure 3 shows a patient who underwent wide excision for basal cell carcinoma located in the left lateral canthal area and RSTL-matched FTSG. In the closed-eye view, wrinkles on the graft site are shown in harmony with the surrounding tissue. However, Figure 4 is a patient who underwent wide excision of basal cell carcinoma located in the infraorbital area and RSTL-unmatched FTSG. In the static phase, the graft site does not look prominent, but in the closed-eye view, the wrinkles do not appear when compared to the contralateral side, so the graft site had a “surgical” appearance. We suspected that the RSTL-matched FTSG was effective in the dynamic phase when the skin was thin and the musculature was close to the wound bed.

We have not demonstrated how much better esthetic and functional results the RTSL-matched FTSG can achieve with facial musculature motion in this study. This is because there has been no discussion on the dynamic scar assessment scale. However, the authors were able to identify natural textures in the RSTL-matched group during facial animation (Figure 3). We believe this is because collagen fibers are arranged in a relatively parallel manner.

**Figure 5:**
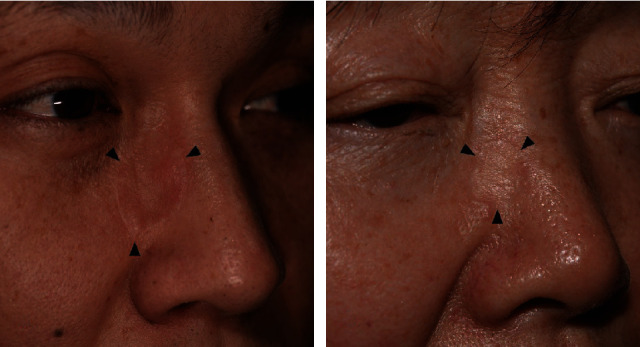
(a) Photograph of RSTL-unmatched patient taken three years postoperatively and (b) photograph of RSTL-matched patient taken three years postoperatively. Compared to the RSTL-unmatched patient, the RSTL-matched patient shows a more natural look with the surrounding tissue.

Figure 5 shows patients undergoing FTSG with wide excision for basal cell carcinoma located on the right nasal sidewall. Patients were followed up three years after surgery.  Figure 5(a) presents a patient who has undergone RSTL-unmatched FTSG, and Figure 5(b) illustrates a patient who has undergone RSTL-matched FTSG. Compared to RSTL-unmatched FTSG, the contour and texture of the RSTL-matched graft are naturally seen with surrounding tissues. Statistical effectiveness could not be demonstrated due to the lack of follow-up observations on sufficient patients, but the author suspected that as time passed and scar maturation occurred, the effect of RSTL-matched graft would be excellent.

Although we successfully covered facial defects with RSTL-matched FTSG, the present study has some limitations to acknowledge. First, it included a small number of cases and used a nonrandomized retrospective design. Thus, selection biases and the presence of confounding factors are unavoidable. Second, further discussion is needed to use the FTSG according to RSTL for areas other than the face. RSTL has many definitions in previous literature [20, 21]. Various skin lines, such as Cox's line and Rubin's line, are defined, including the most frequently mentioned Langer's line. In the face, skin lines are mostly defined similarly, but those of the trunk and extremity are defined differently according to motion and position [22]. This is because tension acts differently depending on the position. Therefore, the author believes that skin grafts according to RSTL can be applied consistently to faces defined by similar skin tension lines.

## 5. Conclusion

A FTSG can be an attractive option when superficial defects are present on the face. When FTSG was performed, matching RSTL in the facial area showed significant scar quality improvement. Although the evaluation method was limited, RSTL-matched FTSG showed a natural appearance due to better assimilation to facial animation. Therefore, when covering superficial defects on the face with FTSG, matching the graft's RSTL along the surrounding RSTL can result in better scar quality and facial esthetics.

## Figures and Tables

**Table 1:** Characteristics of the patients.

Characteristics	*n* (%)
Sex	
Male	5 (23.8)
Female	16 (76.2)
RSTL	
Matched	9 (42.9)
Unmatched	12 (57.1)
Age (yr)	
40~49	1 (4.8)
50~59	1 (4.8)
60~69	3 (14.3)
70~79	6 (28.6)
80~89	7 (33.3)
90~99	3 (14.3)
Defect area (mm^2^)	
100-199	3 (14.3)
200-299	3 (14.3)
300-399	2 (9.5)
400-499	4 (19.0)
500-599	2 (9.5)
>600	7 (33.3)
Type of malignant skin cancer	
Basal cell carcinoma	9 (42.9)
Squamous cell carcinoma	6 (28.6)
Bowen's disease	6 (28.6)
Site of malignant skin tumor	
Cheek	6 (28.6)
Nose	10 (47.6)
Philtrum	1 (4.8)
Temple	4 (19.0)
Location of skin donor site	
Postauricular	7 (33.3)
Supraclavicular	14 (66.7)

**Table 2:** Interobserver reliability of the Vancouver scar scale (VSS) and the observer component of the patient and observer scar assessment scale (OSAS).

Variable	Single measure ICC (95% CI)	Average measure ICC (95% CI)
VSS		
Vascularity	0.362 (0.171~0.596)	0.739 (0.508~0.881)
Pigmentation	0.208 (0.044~0.449)	0.568 (0.186~0.803)
Pliability	0.296 (0.115~0.537)	0.678 (0.393~0.8535)
Height	0.260 (0.084~0.501)	0.637 (0.316~0.834)
Total	0.540 (0.346~0.738)	0.854 (0.726~0.934)
Observer component of the POSAS		
Vascularity	0.480 (0.283~0.693)	0.822 (0.664~0.919)
Pigmentation	0.425 (0.229~0.650)	0.787 (0.598~0.903)
Thickness	0.304 (0.121~0.544)	0.686 (0.408~0.857)
Relief	0.278 (0.099~0.519)	0.658 (0.355~0.844)
Pliability	0.392 (0.199~0.623)	0.764 (0.555~0.892)
Surface area	0.416 (0.222~0.643)	0.781 (0.587~0.900)
Overall	0.506 (0.310~0.713)	0.837 (0.692~0.925)
Total	0.482 (0.285~0.694)	0.823 (0.666~0.919)

**Table 3:** Correlation between the patient component of the POSAS (PSAS) and Vancouver scar scale (VSS) and observer component of the POSAS (OSAS).

	Pearson's correlation coefficient	*p* value
VSS vascularity score vs. PSAS color score	-0.068	0.77
VSS pigmentation score vs. PSAS color score	0.038	0.87
VSS pliability score vs. PSAS stiffness score	0.657	0.001^∗^
VSS height score vs. PSAS thickness score	0.542	0.011^∗^
VSS total score vs. PSAS total score	0.708	≤0.001^∗^
OSAS vascularity score vs. PSAS color score	0.168	0.465
OSAS pigmentation score vs. PSAS color score	-0.081	0.727
OSAS pliability score vs. PSAS stiffness score	0.258	0.259
OSAS thickness score vs. PSAS thickness score	0.069	0.768
OSAS relief score vs. PSAS irregularity score	0.253	0.268
OSAS overall score vs. PSAS overall score	0.129	0.577
OSAS total score vs. PSAS total score	0.363	0.105

**Table 4:** Vancouver scar scale comparison according to RSTL.

	RSTL matched (SD)	RSTL unmatched (SD)	Total (SD)	Mann–Whitney *U*	*p* value
Pigmentation	0.42 (0.35)	0.85 (0.4)	0.67 (0.43)	85.5	0.023^∗^
Vascularity	0.38 (0.46)	0.97 (0.35)	0.71 (0.49)	91	0.007^∗^
Pliability	0.51 (0.32)	1.3 (0.43)	0.96 (0.55)	101	≤0.001^∗^
Height	0.47 (0.49)	0.87 (0.39)	0.7 (0.47)	81.5	0.049^∗^
Total	1.78 (1.12)	3.98 (0.99)	3.04 (1.52)	101	≤0.001^∗^

**Table 5:** Observer scar assessment scale comparison according to RSTL.

	RSTL matched (SD)	RSTL unmatched (SD)	Total (SD)	Mann–Whitney *U*	*p* value
Vascularity	1.40 (0.48)	1.95 (1.29)	1.71 (1.04)	65	0.464
Pigmentation	1.62 (0.61)	2.71 (2.25)	2.25 (1.22)	78.5	0.082
Thickness	1.58 (0.70)	2.03 (0.84)	1.84 (0.80)	74.5	0.148
Relief	1.56 (0.43)	2.12 (0.75)	1.88 (0.68)	78	0.095
Pliability	1.56 (0.41)	2.48 (0.68)	2.01 (0.74)	94.5	0.002^∗^
Surface area	1.58 (0.65)	2.97 (1.07)	2.37 (1.14)	95.5	0.002^∗^
Overall	1.69 (0.70)	2.85 (1.00)	2.35 (1.05)	94	0.003^∗^
Total	10.98 (2.98)	17.12 (5.48)	14.49 (5.46)	95	0.002^∗^

Mann–Whitney *U* test.

**Table 6:** Patient scar assessment scale comparison according to RSTL.

	RSTL matched (SD)	RSTL unmatched (SD)	Total (SD)	Mann–Whitney *U*	*p* value
Painful	1 (0)	1.42 (0.51)	1.24 (0.44)	76.5	0.111
Itching	1.22 (0.67)	1.25 (0.45)	1.24 (0.54)	60	0.702
Color	1.78 (0.83)	1.83 (0.83)	1.81 (0.81)	56	0.917
Stiffness	1 (0)	1.92 (0.79)	1.52 (0.75)	90	0.009^∗^
Thickness	1.11 (0.33)	2.67 (0.98)	2 (1.1)	100.5	≤0.001^∗^
Irregularity	1.44 (0.73)	2.58 (0.79)	2.1 (0.94)	91	0.007^∗^
Overall	1.33 (0.5)	2.25 (0.97)	1.86 (0.91)	88.5	0.012^∗^
Total	8.89 (1.54)	13.92 (2.02)	11.76 (3.11)	108	≤0.001^∗^

Mann–Whitney *U* test.

## Data Availability

The datasets and/or analysis results used during the current study are available from the corresponding authors upon reasonable request.

## Additional Points

*Key Messages*. (i) A FTSG can be an attractive option when superficial defects are present on the face. (ii) When FTSG was performed, matching RSTL in the facial area could provide significant scar quality improvement.

## Supplementary Materials

Supplementary Materials Supplementary 1: patient and observer scar assessment scale. Supplementary 2: Vancouver scar scale.
