# Supplementary material for: Full-Thickness Skin Graft according to Surrounding Relaxed Skin Tension Line Improves Scar Quality in Facial Defect Coverage: A Retrospective Comparative Study
Source: Biomed Res Int. 2021 Sep 15;2021:7398090. doi: 10.1155/2021/7398090 (PMC8460372; doi:10.1155/2021/7398090)
Supplement: Supplementary Materials — Supplementary 1: patient and observer scar assessment scale. Supplementary 2: Vancouver scar scale. [file 7398090.f1.docx]

| **Observer component** | **Normal skin** | | | | | | **Worst scar imaginable** | | | | |
| --- | --- | --- | --- | --- | --- | --- | --- | --- | --- | --- | --- |
|  | 1 | 2 | 3 | 4 | 5 | 6 | | 7 | 8 | 9 | 10 |
| Vascularity | O | O | O | O | O | O | | O | O | O | O |
| Pigmentation | O | O | O | O | O | O | | O | O | O | O |
| Thickness | O | O | O | O | O | O | | O | O | O | O |
| Relief | O | O | O | O | O | O | | O | O | O | O |
| Pliability | O | O | O | O | O | O | | O | O | O | O |
| Surface area | O | O | O | O | O | O | | O | O | O | O |
| Overall opinion | O | O | O | O | O | O | | O | O | O | O |
| Patient component | No | | | | | | Yes | | | | |
|  | 1 | 2 | 3 | 4 | 5 | 6 | | 7 | 8 | 9 | 10 |
| Is the scar painful? | O | O | O | O | O | O | | O | O | O | O |
| Is the scar itching? | O | O | O | O | O | O | | O | O | O | O |
| Is the color of the scar different? | O | O | O | O | O | O | | O | O | O | O |
| Is the scar more stiff? | O | O | O | O | O | O | | O | O | O | O |
| Is the thickness of the scar different? | O | O | O | O | O | O | | O | O | O | O |
| Is the scar irregular? | O | O | O | O | O | O | | O | O | O | O |
| Overall opinion | O | O | O | O | O | O | | O | O | O | O |

**Supplementary 1. Patient and Observer Scar Assessment Scale**

**Supplementary 2. Vancouver Scar Scale**

| **Skin characteristics** | **Parameters** |
| --- | --- |
| **Pliability** |  |
| 0 | Normal |
| 1 | Supple |
| 2 | Yielding |
| 3 | Firm |
| 4 | Ropes |
| 5 | Contracture |
| **Height** |  |
| 0 | Flat |
| 1 | <2mm |
| 2 | 2-5mm |
| 3 | >5mm |
| **Vascularity/erythema** |  |
| 0 | Normal |
| 1 | Pink |
| 2 | Red |
| 3 | Purple |
| **Pigmentation** |  |
| 0 | Normal |
| 1 | Hypopigmented |
| 2 | Mixed |
| 3 | Hyperpigmented |
